# Offspring Hormones Reflect the Maternal Prenatal Social Environment: Potential for Foetal Programming?

**DOI:** 10.1371/journal.pone.0145352

**Published:** 2016-01-13

**Authors:** Kristine Meise, Nikolaus von Engelhardt, Jaume Forcada, Joseph Ivan Hoffman

**Affiliations:** 1 Department of Animal Behaviour, University of Bielefeld, Morgenbreede 45, 33615, Bielefeld, Germany; 2 British Antarctic Survey, High Cross, Madingley Road, Cambridge, CB3 OET, United Kingdom; Sonoma State University, UNITED STATES

## Abstract

Females of many species adaptively program their offspring to predictable environmental conditions, a process that is often mediated by hormones. Laboratory studies have shown, for instance, that social density affects levels of maternal cortisol and testosterone, leading to fitness-relevant changes in offspring physiology and behaviour. However, the effects of social density remain poorly understood in natural populations due to the difficulty of disentangling confounding influences such as climatic variation and food availability. Colonially breeding marine mammals offer a unique opportunity to study maternal effects in response to variable colony densities under similar ecological conditions. We therefore quantified maternal and offspring hormone levels in 84 Antarctic fur seals (*Arctocephalus gazella*) from two closely neighbouring colonies of contrasting density. Hair samples were used as they integrate hormone levels over several weeks or months and therefore represent *in utero* conditions during foetal development. We found significantly higher levels of cortisol and testosterone (both *P* < 0.001) in mothers from the high density colony, reflecting a more stressful and competitive environment. In addition, offspring testosterone showed a significant positive correlation with maternal cortisol (*P* < 0.05). Although further work is needed to elucidate the potential consequences for offspring fitness, these findings raise the intriguing possibility that adaptive foetal programming might occur in fur seals in response to the maternal social environment. They also lend support to the idea that hormonally mediated maternal effects may depend more strongly on the maternal regulation of androgen rather than cortisol levels.

## Introduction

In mammalian species, most of the development of all major organs occurs during the prenatal phase, making the foetus particularly vulnerable to external influences. Thus, it has been proposed that environmental stimuli during critical periods may alter foetal development, with important implications for the offspring later in life (the 'foetal programming hypothesis', [1,2]). This concept was initially developed around the discovery that people with lower birth weights tend to be more susceptible to coronary heart disease, suggesting that exposure of the foetus to adverse conditions *in utero* may permanently alter tissue structure and function [3,4].

Originally, foetal programming was viewed as a disruptive process, with mainly negative implications for the offspring. However, there is growing interest and support for the idea that foetal programming might also be adaptive [5,6]. Thus, if maternal conditions reflect a stable and predictable environment that the offspring is likely to encounter after birth, alterations in developmental processes in relation to these conditions may be beneficial to offspring survivorship and could even propagate into adulthood, conferring greater survival and reproductive success [7].

Hormones function as chemical messengers that trigger physiological and behavioural responses to a variety of stimuli. Consequently, maternal hormones could play an important role in foetal programming, as they can cross the placenta and thereby influence the development of the foetus. Decades of research in humans and animal model species have uncovered considerable empirical support for this notion. A large number of studies on foetal programming focus on androgens and corticosteroids, which affect a broad range of physiological processes such as metabolism, growth, reproduction, the immune system and the nervous system [8–10]. Whether these have positive or negative consequences for survival and reproduction depends upon the environmental context and internal state of the animal. In humans, for example, elevated maternal cortisol levels alter the activity of the hypothalamic-pituitary adrenal (HPA) axis of the foetus, leading to elevated stress responses in the children of stressed mothers (reviewed in [11]). Moreover, a large body of evidence in animals points towards the prenatal administration of testosterone triggering the 'masculinization' of offspring (e.g. [12,13]).

More recently, a number of studies have revealed evidence for potentially adaptive developmental changes shaped by maternal hormones. For example, in the three-spined stickleback (*Gasterosteus aculeatus*), mothers that are exposed to the threat of predation produce larger eggs containing more cortisol. Their offspring in turn show tighter shoaling behaviour, which is believed to be an adaptive response to increased predation risk [14]. Thus, maternal hormones appear capable of 'forecasting' environmental conditions after birth and triggering physiological and / or behavioural changes that may help to match offspring to the environment to which they will be exposed [7,15].

Social density can have strong effects on the endocrine system [16,17]. For example, high population density can lead to increased competition for resources such as food, breeding habitat and access to mates, thereby increasing the levels of 'classical' stress hormones such as cortisol [18–21]. Similarly, testosterone levels are often elevated under high density conditions, as they tend to correlate positively with the frequency and intensity of agonistic interactions [22]. Overall, it could be argued that these hormonal changes are likely to be adaptive, in the sense that higher levels of both cortisol and testosterone should allow individuals to be more competitive under stressful conditions.

Many species can be found in populations that differ in density, either geographically or over time. This begs the question of whether foetal programming mediated by maternal hormones could play a role in adapting offspring to contrasting social conditions. Probably the best example for adaptive foetal programming in a natural population comes from a long-term study of North American red squirrels, *Tamiasciurus hudsonicus*. Here, high maternal cortisol levels in response to increased social density have been shown to increase offspring growth rates, and faster growth in turn increases the probability of juvenile overwinter survival [23]. However, it is usually difficult to assess the impact of social density on hormone levels in a natural setting due to potentially confounding influences of other variables. For example, increased competition for food in high density populations can lead to nutritional stress, which in turn is associated with higher cortisol levels [24,25].

Females of many pinniped species are highly gregarious and aggregate in crowded breeding colonies, which are usually located in remote and inaccessible sites such as oceanic islands [26]. Population density can vary markedly among colonies due to factors such as topology, breeding habitat quality and proximity to foraging grounds [27,28]. However, established colonies tend to be relatively stable over space and time, as females are highly philopatric [29]. Moreover, there are many locations in which breeding colonies of different densities can be found in such close proximity that they share the same foraging grounds [30] and thus should be affected equally by spatiotemporal variation in food availability. Pinnipeds therefore provide an unusual opportunity to investigate maternal effects on foetal development while controlling for potentially confounding variables such as food availability.

Antarctic fur seals (*Arctocephalus gazella*) can be found on most of the subantarctic islands, but the majority of the population breeds around South Georgia [31]. Females of this species aggregate on land during a short breeding season in the Austral summer (November to January). Shortly after coming ashore, they give birth to a single pup conceived the previous season, which they suckle continuously for up to seven days [32,33]. They subsequently alternate provisioning bouts ashore with foraging trips at sea, before the pup is weaned at around four months of age [34]. Females usually moult during lactation or immediately after the weaning of the pup. Afterwards, the animals go to sea where they spend the Austral winter foraging [35].

Antarctic fur seals provide a potential candidate for foetal programming in relation to social density for a number of reasons. First, adult females return to breed to within a body length of where they were born [36] and are also highly site-faithful as adults [37]. This means that female pups born in a particular colony are likely to breed in the same colony later in life, and thus that offspring should be exposed to a relatively predictable social environment. Second, breeding female densities vary from around 1.5 females / m^2^ at high density sites to around 0.2 females / m^2^ at low density sites [38] and this has consequences for both adults and pups. In particular, pup mortality is density dependent, being around five to ten times greater in high density colonies, mainly due to an increase in traumatic injuries caused by female bites to the head and male trampling [39–41]. Also, genetic studies of a high density colony at Bird Island, South Georgia, and a low density colony at Cape Shirreff, Livingstone Island, suggest that social density can have a profound influence on the mating system [42]. While polygyny operates at both colonies, the low density site is characterized by far greater male reproductive skew, suggesting that the most successful males are better able to monopolize access to breeding females.

**Fig 1:**
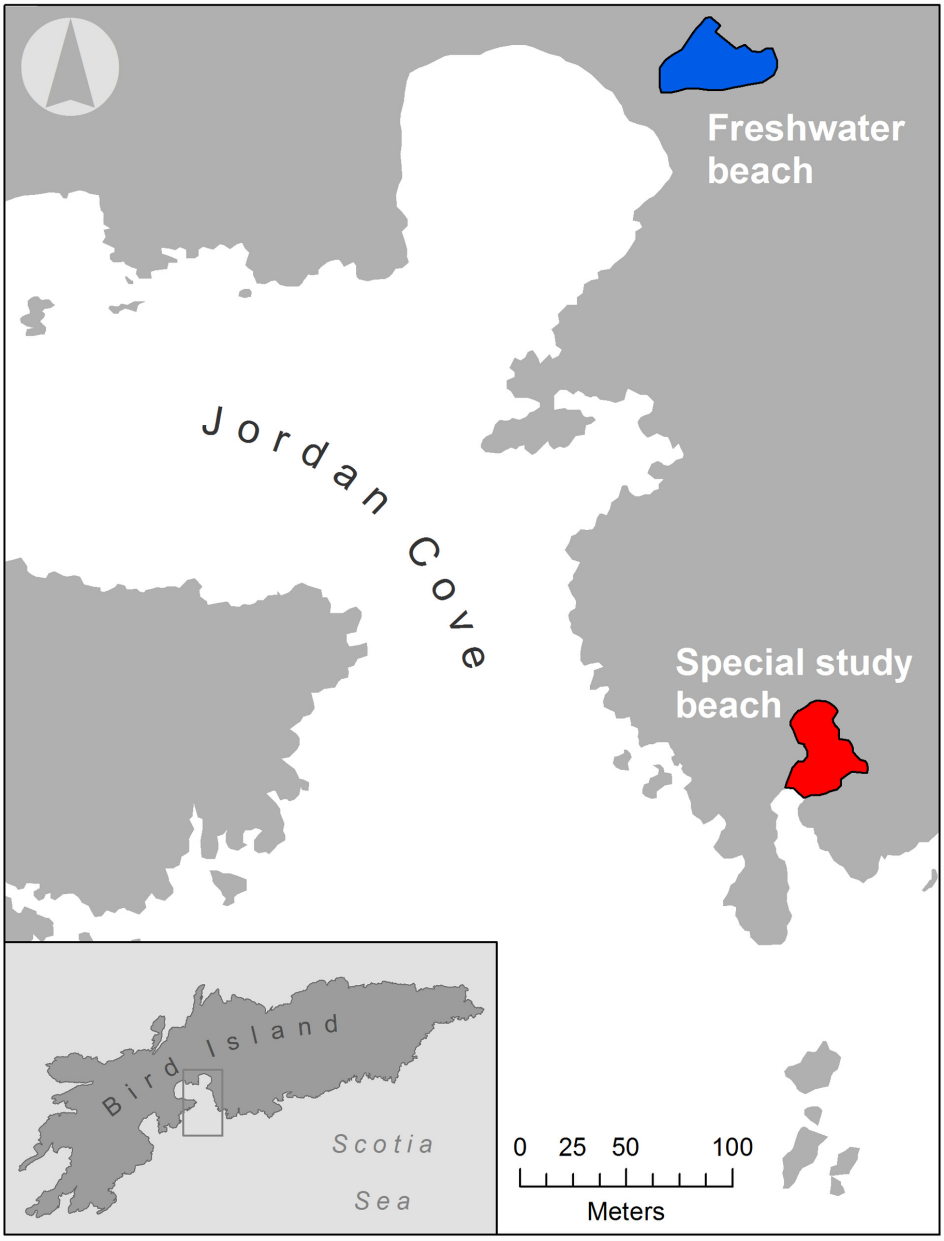
Map showing locations of the high density (red) and low density (blue) Antarctic fur seal breeding colonies used for this study on Bird Island, South Georgia.

On Bird Island, two fur seal colonies of contrasting density are situated just 200m apart (Fig 1). As breeding females from these neighbouring colonies are subject to identical climatic conditions and share the same foraging grounds, this provides a convenient system in which to investigate the effects of social density on hormone levels and to test for maternal effects on offspring hormone levels. Moreover, the two colonies are not genetically different from one another [43] allowing us to exclude the possibility that any hormonal differences between them could be associated with population structure. Consequently, we quantified cortisol and testosterone in the hair of mother-offspring pairs from these two colonies. Hair was used in preference to blood samples because hair integrates hormone levels over several months and therefore captures information about the hormonal environment during foetal development [44–46]. We hypothesized that females breeding in the high density colony would have higher levels of both cortisol and testosterone. As a prerequisite for foetal programming in response to social density, we also expected offspring hormone levels to be density-dependent and to correlate positively with the levels of maternal hormones.

Finally, previous studies of fur seals at South Georgia have uncovered an important component of individual quality in the form of multilocus heterozygosity [47–49]. Individual heterozygosity has also been shown to correlate with aggressiveness and social dominance in other vertebrate species [50,51] but has rarely been analysed in relation to hormone levels. We therefore quantified each individual’s heterozygosity at 41 highly polymorphic microsatellite loci and used the resulting data to test for associations between heterozygosity and hormone levels.

## Methods

### Study area

The study was conducted in December 2011 at two breeding beaches on Bird Island, South Georgia (54° 00´ S, 38° 02´ W). Although population sizes fluctuate from year to year [49], census data from 2010–2015 indicate that the high density colony has a mean density 4.02 ± 0.48 SE times higher than the low density colony (high density colony = 1.15± 0.11 SE animals / m^2^; low density colony = 0.29 ± 0.02 SE animals / m^2^). From the low density colony at Freshwater beach, we focused on an area of approximately the same size as the high density colony, which is demarcated in Fig 1.

The sampling of animals at both colonies was randomized with respect to location and timing during the pupping season. While we do not have age data for females from Freshwater beach, standard length and axillary girth measurements of the sampled seals do not show significant differences between beaches, suggesting a similar demographic structure. Both study colonies have been worked on consistently over the last three decades and therefore any human disturbance, which is unlikely to be significant, should also be consistent across colonies and through time. The high return rates of seals tagged on both beaches over the years indicates that the long-term consequences of disturbance are minimal.

### Animal handling and sample collection

Breeding females and their pups were captured and restrained on land using standard methodology (for a detailed description, see [52]). Briefly, adult females were captured with a noosing pole and transferred to a restraint board for processing. Afterwards they were released as close as possible to her initial capture site, taking care to ensure that they return to their pup with a minimum of disturbance. Pups were captured with a slip noose and returned to their mothers as quickly as possible. Seal capture and restraint were part of annual routine procedures of the Long Term Monitoring and Survey Programme of the British Antarctic Survey.

A small sample of fur (< 100 mg) was taken from the lower back of each individual (high density colony = 17 mother-offspring pairs, low density colony = 25 pairs). The hair was cut with scissors as close to the skin as possible and each sample was dried and transferred to a paper envelope, which was then sealed and stored in the dark. Tissue samples were also collected from each animal as described by Hoffman et al. [53] and stored individually at –20°C in the preservative buffer 20% dimethyl sulphoxide (DMSO) saturated with salt. Females were randomly selected with respect to age and all sampling was conducted within 2–3 days of the birth of pups. Each mother was sampled on the same day as her pup.

### Genetic analysis

Total genomic DNA was extracted from each sample using a standard phenol-chloroform protocol and genotyped at 41 highly polymorphic microsatellite loci (see S1 Table for details). These were PCR amplified in eight separate multiplexed reactions using a Type It Kit (Qiagen). The following PCR profile was used: one cycle of 5 min at 94°C; 24 cycles of 30 s at 94°C, 90 s at Ta°C and 30 s at 72°C; and one final cycle of 15 min at 72°C (see S1 Table for details). Fluorescently labelled PCR products were then resolved by electrophoresis on an ABI 3730xl capillary sequencer and allele sizes were scored automatically using GeneMarker v1.95. To ensure high genotype quality, all of the genotypes were manually inspected and any that had been incorrectly determined by the program were adjusted accordingly. As measures of each individual's genetic quality, we calculated homozygosity weighted by locus (HL) [54] and standardized multilocus heterozygosity (sMLH) [55].

### Hormone assays

15–30 mg of each hair sample was washed twice for one minute with 1ml isopropanol in order to remove any contaminants from the external surface of the hair shaft. The amount of residual cortisol measured after an additional third wash was minimal (0.25ng/g compared to ~2ng/g after the first two washes, as also shown by Davenport et al. [45]). The hair was then dried overnight at room temperature under a fume hood and ground to a fine powder using a compact bead mill (5 min. at 50Hz, TissueLyzer LT, Quiagen). For the hormone extraction, 600μl of methanol was added and each sample was incubated at 45°C for 18h with gentle shaking. The samples were then centrifuged for 5 min at 6600 g and the supernatant extract was transferred with a pipette to a new tube for subsequent hormone analysis. The hair powder was rinsed twice with 200μl of methanol, each time centrifuging as before, and the resulting supernatants were combined (following [46]). The extract was then passed through a small plug of filter paper (MN 615, Macherey-Nagel GmbH and Co. KG, Germany) in a pipette tip to remove any remaining fragments of hair. Subsequently, the methanol extract was desiccated within 2 h at 45°C under a stream of nitrogen gas, and the sample was reconstituted in 450μl PBS buffer, homogenised by vortexing for 30 sec, and stored at -10°C prior to measurement.

To quantify cortisol and testosterone concentrations in the hair samples, we used enzyme-linked immune sorbent assays (ELISAs) developed for the quantitative measurement of active free cortisol and testosterone in saliva (DES6611 and DES6622, Demeditec Diagnostics GmbH, Germany). ELISAs were carried out in accordance to the manufacturer's protocols. Cross-reactivity of the antibodies in the cortisol assay was found with Prednisolone (63.4%), 11-Deoxycortisol (10.4%), Corticosterone (5.2%) and all other tested steroids (≤ 1%). The antibodies of the testosterone assay had the following cross reactivity: 5α-Dihydrotestosterone 23.3%, Androstenedione 1.6% and all other tested hormones ≤ 0.1%. The intra-assay coefficients of variation for cortisol and testosterone were 5.5% and 16.4% respectively.

Two samples (one mother and one pup from the high density colony) showed a yellowish colour after extraction which seemed to affect the hormone measurements as they were beyond the upper detectable limit of the cortisol assay and close to the detection limit of the testosterone assay. Therefore, we decided to exclude these samples from further analysis. In addition, we excluded one sample from a pup from the low density colony from the cortisol analysis with a hormone level beyond the upper detectable limit of the assay. Further, we were missing testosterone data for two mother-pup pairs, one from each colony, due to a shortage of assay space. In Table 1 we briefly summarize the samples sizes used for statistical analysis. No correlation was found between the amount of hair used for the extraction and the amount of hormone per mg hair measured (Pearson correlation, cortisol: *N* = 81, *r* = -0.18, *P* = 0.11, testosterone: *N* = 78, *r* = 0.17, *P* = 0.14).

**Table 1:** Sample sizes used for statistical analysis.

	Cortisol		Testosterone	
	Low density	High density	Low density	High density
Mothers	25	16	24	15
Offspring	24 (♀ 9 ♂ 15)	16 (♀ 8 ♂ 8)	24 (♀ 10 ♂ 14)	15 (♀ 7 ♂ 8)
Total	49	32	48	30

Sample sizes include all individuals for which cortisol and testosterone measurements could be obtained from high and low density Antarctic fur seal breeding colonies at Bird Island, South Georgia. The total sample sizes indicate the numbers of mothers plus the numbers of offspring.

### Statistical analysis

For the statistical analyses, hormone concentrations were log-transformed in order to meet normality assumptions. A linear model (LM) was used to assess how testosterone levels differed with regard to colony density (fitted as a two level factor, coded as 1 = high and 0 = low density), age class (fitted as a two level factor, coded as 1 = mother and 0 = offspring) and their interaction as well as multilocus heterozygosity (measured as HL or sMHL and fitted as a continuously distributed variable). To control for any possible temporal effects, we included date as a continuous variable. For cortisol, we used the same set of factors, but a robust-resistant regression model (RLM [56]) as the LM violated the assumption of normality due to the presence of a single outlier (female 18, S2 Table). Due to the same outlier, we also used an RLM to assess how maternal cortisol relates to maternal testosterone, fitting as predictor variables maternal testosterone and colony density as well as the interaction between both variables. Finally, we constructed a series of LMs of offspring cortisol and testosterone, fitting either density, sex, maternal cortisol and the interaction between density and maternal cortisol as predictors, or density, sex maternal testosterone and the interaction between density and maternal testosterone as predictors. All of the models we fitted are summarised in Table 2.

**Table 2:** Summary of the results of seven different models that were implemented to identify variables associated with hormone levels in fur seal mothers and pups.

Model	Response variable	Fixed effects	Sample size
1	cortisol	Age * density * heterozygosity + date	81
2	testosterone	Age * density * heterozygosity + date	78
3	Maternal cortisol	Density * maternal testosterone	37
4	Pup cortisol	Density * maternal cortisol + sex	37
5	Pup cortisol	Density * maternal testosterone + sex	37
6	Pup testosterone	Density * maternal cortisol *+ sex*	37
7	Pup testosterone	Density * maternal testosterone + sex	37

Besides model 1 and 3 which were robust-resistant models, all models were linear models. Model selection was based on a maximum likelihood approach (see Methods for details). Fixed effects that were retained in the models are shown in normal style and those dropped from the models are shown in italics. Furthermore, to explore possible maternal effects, we focused on 37 mother-offspring pairs for which data on both cortisol and testosterone data were available (models 3–7).

Model selection was based on the maximum likelihood approach, which allows direct comparison of models with different fixed effect structures [57,58]. Fixed factor estimates were obtained by refitting the best model with a restricted maximum likelihood method [58]. All statistical analyses were carried out using R v.3.1.3 [59]. Differences were considered significant at *P* < 0.05. Hormone levels are presented as means ± SE.

#### Ethical note

Fur samples were collected by one of the authors (JF) as part of the Long Term Monitoring and Survey project of the British Antarctic Survey that has employed consistent sampling protocols since 1994. Sampling was authorised by the Senior Executive and the Environment Officers of the Government of South Georgia and the South Sandwich Islands, and samples were collected under Scientific Research Permits for the British Antarctic Survey field activities on South Georgia during the 2010/11 seasons. All procedures used were approved the British Antarctic Survey Ethics Committee (reference number PEA6).

## Results

Considerable variation was found in the hormone levels of individual fur seals, with cortisol concentrations ranging from 8.3 to 164.4 pg/mg and testosterone concentrations ranging from 0.5 to 56.3 pg/mg. Most of this variance could be explained by the density of the breeding colony and the difference between mothers and offspring (cortisol: Model 1, adjusted *R*^*2*^ of model = 0.79; testosterone: Model 2, adjusted *R*^*2*^ of model = 0.52).

**Fig 2:**
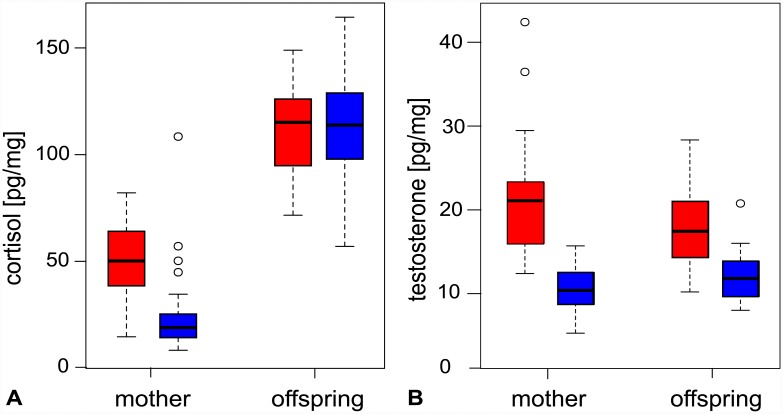
Boxplots showing variation in (a) cortisol and (b) testosterone in fur seal mother-offspring pairs from the high density (red) and low density (blue) breeding colony. These graphs are based on the raw (i.e. non-transformed) data.

**Fig 3:**
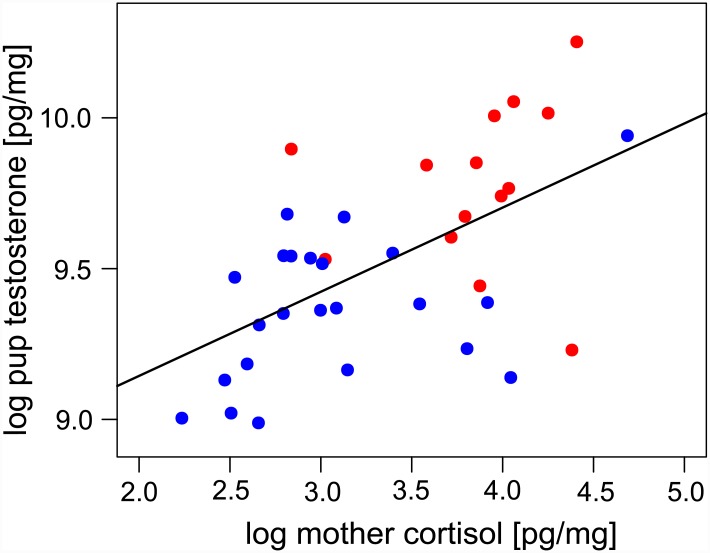
Relationship between pup testosterone and maternal cortisol in the high density (red) and low density (blue) breeding colonies. This is based on 37 mother-offspring pairs for which data on both cortisol and testosterone data were available. Points represent the log transformed data analyzed in the model and the line shows the model output.

Adult females that gave birth in the high density colony had significantly higher cortisol levels than those in the low density colony (high: 50.0 ± 20.9, low: 25.6 ± 21.3, Fig 2a; Model 1, Table 2; *t* = 8.50, *P* <0.001). The same pattern was also reflected in the testosterone levels of mothers (high: 24.8 ± 11.9, low: 10.6 ± 2.6, Fig 2b, Model 2, Table 2; *t* = 8.35, *P* < 0.001). Furthermore, a significant positive correlation was observed between maternal cortisol and testosterone that was unaffected by colony density (Fig 3, Model 3, Table 2; *t* = 8.22, *P* < 0.001).

Pups had significantly higher cortisol levels than adult females (Fig 2a, Model 1, Table 2; *t* = 18.22, *P* < 0.001). Cortisol levels did not differ significantly between pups born in high and low density colonies (high: 111.0 ± 21.9, low: 111.2 ± 24.5, Fig 2a, Model 1, Table 2; *t* = -0.24, *P* > 0.05) and accordingly were uncorrelated with maternal hormone levels (cortisol: Model 4, Table 2; *t* = 0.52, *P* = 0.61; testosterone: Model 5, Table 2; *t* = 0.23, P = 0.82). In contrast, pup testosterone levels were elevated in the high density colony (high: 17.9 ± 4.8, low: 12.1 ± 3.0, Fig 2b; Model 1, Table 2; *t* = 4.10, *P* < 0.001) and correlated positively with maternal cortisol (Fig 3; Model 6, Table 2; *t* = 2.03, *P* < 0.05) but not with maternal testosterone (Model 7, Table 2; *t* = 0.38, *P* = 0.70). Neither hormone differed significantly between male and female pups (cortisol: Model 6, Table 2; *t* = 0.31, *P* = 0.76; testosterone: Model 7, Table 2; *t* = -0.17, *P* = 0.87).

Finally, we tested for associations between individual multilocus heterozygosity, estimated from 41 microsatellite loci, and hormone levels. Neither HL nor sMLH were significantly correlated with cortisol (HL: Model 1, Table 2; *t* = 0.82, *P* = 0.42; sMLH: Model 1, Table 2; *t* = -0.74, *P* = 0.46) or testosterone (HL: Model 2, Table 2; *t* = -1.56, *P* = 0.12; sMLH: Model 2, Table 2; *t* = 1.31, *P* = 0.196) indicating that heterozygosity, a measure of genetic quality, does not appear to be associated with hormonal status.

## Discussion

Colonially breeding marine mammals offer a unique opportunity to study maternal effects in response to highly variable colony densities under similar ecological conditions. We therefore quantified maternal and offspring hormone levels in two neighbouring fur seal colonies that differ in social density but are otherwise exposed to ecologically equivalent conditions. We found that cortisol and testosterone levels were both significantly elevated in mothers from the high density colony, whereas in pups only testosterone showed a density-dependent pattern. Furthermore, maternal cortisol was significantly correlated with offspring testosterone regardless of colony. Although further work will be needed to explore any potential consequences for offspring fitness, this correlation provides insights into how female fur seals could potentially program their pups in relation to social density.

The majority of studies of foetal programming have so far been conducted in the laboratory. By experimentally altering environmental conditions at precise time-points during pregnancy, these studies have yielded insights into the timing and consequences of developmental processes in the foetus (e.g. [7,60,61]). However, it is difficult to generalize the results of laboratory studies to ecologically relevant contexts due to a paucity of data from natural populations (although see [23] and [62]). Moreover, in many natural situations, the effects of a given environmental factor cannot easily be disentangled from those of other confounding variables. Our study design allowed us to investigate the effect of social density on maternal and offspring hormone levels while controlling as far as possible for any potentially confounding effects of climate and food availability.

### Density-dependent differences in maternal hormones

We found significantly higher levels of both cortisol and testosterone in adult females from the high density colony. As hormone levels were quantified from fur, these differences reflect reasonably long-term or chronic circulating levels and not short-term (i.e. hourly or daily) fluctuations. Such a pattern could conceivably result from spatial and temporal segregation of individuals according to age, breeding experience and competitiveness. Younger females tend to pup later than more experienced breeders and for that reason are less likely to find optimum territories [63], which will already have been occupied by experienced females [36]. On the other hand, more competitive and experienced males tend to hold territories on prime locations, which are typically closer to the water’s edge [64]. However, given the lower density of Freshwater beach and the fact that access to any available seal is also possible on the special study beach, we implemented a randomized sampling design with respect to location and timing during the pupping season. This is reflected in the fact that the biometrics of the sampled seals did not differ significantly between the two colonies.

Similar patterns have been reported in several other species, both in natural and experimental settings (e.g. [19–21,65]). For example, Dettmer et al. [20] showed that cortisol levels in rhesus monkeys fluctuated over a five year period in relation to social density, and Pearson et al. [21] demonstrated that experimentally induced crowding was associated with higher levels of salivary cortisol in baboons. However, very few studies have quantified both cortisol and testosterone simultaneously. Elevated cortisol and testosterone levels are thought to help individuals to cope with increased competition and social stress by mobilizing energy and increasing both activity and competitiveness [17,66]. Thus, female fur seals breeding in crowded fur seal colonies might conceivably benefit from higher levels of these two hormones as they must not only compete for access to breeding space but also cope with harassment from territorial males.

An alternative explanation that we cannot discount, but which seems unlikely, is that parasites and not social stress could be responsible for increased cortisol levels in the high density colony. This is because cytokines, which are produced in response to parasitic infections, are also known to activate the HPA axis and thereby increase cortisol levels (reviewed in [67]). However, immune activation due to an acute infection suppresses testosterone levels [68], leading to the prediction that cortisol and testosterone levels should be negatively correlated. We find the opposite, suggesting that social stress rather than parasite load may be responsible for the differences in hormone levels between the two colonies. To confirm this experimentally would require parasites to be sampled from animals from the two colonies. However, this is not straightforward because it is impractical to collect faeces from the special study beach and therefore animals would need to be sedated in order to access intestinal parasites.

Another possible explanation for our findings could be that animals from the two colonies differ in the extent to which they are nutritionally stressed. We feel this is unlikely to be important as the two colonies are situated just a couple of hundred metres apart, which is a negligible distance for a highly vagile marine mammal. Data from animals instrumented from Freshwater beach also show that the main foraging grounds are not only fairly large, but they are also situated fairly close to the north west of South Georgia [69]. At this point we cannot say definitively that animals from the two colonies forage in the same area, as the special study beach is a long-term monitoring site and therefore we cannot instrument the animals with telemetry devices. At the level of the individual, nutritional stress would be expected to affect steroid hormone levels, but we have no data to explore this possibility and it seems very unlikely that this could explain any differences between closely neighbouring colonies. One way to tackle this question in the future might be through ‘nutrigenomics’, the use of gene expression data from peripheral blood to assess an animal’s nutritional status [70].

Another factor that could potentially influence hormone levels is genetic quality. Multilocus heterozygosity is positively correlated with aggressive behaviour and social status in a number of vertebrate species [50,51,71]. As these traits are to some extent under hormonal control, it is therefore possible that hormone levels could be influenced by heterozygosity. In particular, given that high testosterone levels can lead to increased aggression, one might expect concentrations of this hormone to be positively correlated with heterozygosity. Conversely, a negative association would be expected between cortisol and heterozygosity, as poor quality, relatively homozygous individuals should be more stressed than high quality, relatively heterozygous individuals [72]. However, neither HL nor sMLH were retained in any of our statistical models and, in contrast to the above predictions, maternal cortisol and testosterone were positively correlated. This suggests that hormone levels are unrelated to heterozygosity, although we cannot rule out the possibility of genetic effects being too weak to be detected with our study design.

### Correlations between maternal and offspring hormones

Offspring cortisol levels were on average around twice as high as those of their mothers, a pattern consistent with previous studies of other species based on hair (e.g. [73,74]). This is to be expected as foetal cortisol levels are known to rise steeply during late pregnancy and peak around the time of parturition, reflecting the important role of this hormone in the later developmental stages of many organs and in the activation of specific brain regions [60,75,76]. Offspring cortisol levels also showed no density dependence and were uncorrelated with maternal cortisol levels. This is in contrast to several other species where hair cortisol levels have been shown to correlate positively between mothers and offspring [46,77,78] and reflects species-specific differences in hair growth and replacement. In particular, hormone levels in the hair of many species will reflect hormonal status during late pregnancy when cortisol levels are very high. In contrast, cortisol levels in the hair of fur seal mothers more likely reflect circulating hormones around the period of moult and early pregnancy, whereas offspring hair is more likely to reflect late pregnancy. This could explain why cortisol levels are uncorrelated between mothers and offspring, in a similar way to humans where cortisol levels in offspring hair are correlated with cortisol in maternal hair collected during late but not early pregnancy [78]. An alternative explanation could be that the mammalian placenta normally buffers exposure of the foetus to maternal cortisol by producing 11-β-hydroxysteroid dehydrogenase type 2, an enzyme that converts maternal cortisol into inactive cortisone [79]. Thus, it is possible that maternal cortisol might only be transferred to the offspring when levels are very high such as under conditions of extreme stress [80].

In contrast, offspring testosterone levels showed significant differences in relation to colony density and were positively correlated with maternal cortisol levels. As cortisol is to some extent buffered by the placenta, the underlying causes for the correlation we observe are probably diverse and may involve multiple hormonal pathways (see [7,8,81]). Possible candidates are androgens produced by the maternal adrenal gland, which have been found to increase in parallel to cortisol after exposure to a stressor and can cross the placenta [82,83].

### Potential for adaptive foetal programming

Our study reveals a clear relationship between maternal cortisol and offspring testosterone. Although we have insufficient data to explore the consequences of such a relationship for offspring fitness, we could envisage a number of potential routes by which individuals could be affected, either as pups or later in life. First, studies of other mammals and birds have shown that high prenatal testosterone levels can lead to a more rapid morphological and behavioural development, increased locomotory activity, faster growth and increased competitiveness (e.g. [9,84,85]). Several of these traits could potentially be beneficial to offspring survival under conditions of high social density, where traumatic injuries inflicted by adult females and trampling by adult males are common causes of pup mortality [39–41].

Second, one could also speculate on the potential effects of foetal programming during adulthood. Several studies of other vertebrate species have shown that exposure to increased maternal corticosteroid and androgen levels can modify adult aggressiveness, sexual traits, timing of sexual maturation and dispersal [7,10,86]. Modification of these traits might therefore benefit animals breeding in high-density environments, where agonistic interactions will be stronger and more frequent, and where delayed reproduction may be advantageous by allowing individuals to muster the resources they need to be competitive. This provides an interesting avenue for future research.

### Why do fur seals breed in high density colonies?

We show that maternal stress hormones are elevated under conditions of high social density, despite females having been selected at random from both colonies. This is consistent with pup mortality showing strong density dependence [39–41] and raises the question of why females should choose to breed in high density colonies. One possible explanation is that a female's choice of breeding site might not necessarily be adaptive, but rather a consequence of strong natal philopatry [36]. Alternatively, Bartholomew [26] argued that females may breed in dense aggregations partly to avoid being mated by males of inferior quality who are excluded from prime territories (the 'marginal male hypothesis'). Whether or not the costs of breeding in high density colonies could be offset by potential benefits such as gaining higher quality mates remains to be tested.

### Using hair to assess prenatal hormone exposure

In the present study, hormone concentrations were measured from hair rather than blood. From a practical standpoint, hair can be collected easily and non-invasively. More importantly, levels of cortisol and testosterone in blood plasma are known to change within minutes in response to external stimuli [87–89] and can therefore be affected by animal capture and handling as well as by other stimuli that may operate over short timescales. In contrast, hair integrates hormone levels over an extended time period [45,46,90,91] and thereby provides a window on the conditions experienced during foetal development.

Previous studies of other vertebrate species suggest that hormones in the hair reflect the period of hair growth, a time window of about two to six months prior to hair sampling [45,90,92]. However, most of these species have continuous hair growth, whereas fur seals moult at least part of the underfur and most of the guard hairs during lactation and around the time of weaning [93,94]. Therefore, hormones are most likely incorporated into the maternal hair during lactation and towards the end of the breeding season. Environmental conditions during this period seem to have a strong impact on embryonic development, which is likely linked to maternal hormonal status [63, 95–97].

Two other (non-mutually exclusive) possibilities cannot be discounted at present. First, adult females that breed in high density colonies might have constitutively high hormone levels due to chronic exposure to social stress over multiple successive breeding seasons. This could affect their circulating hormone levels throughout the year, including times when they are not in the colony. A second possibility is that an adult female's endocrine system may have been programmed by the social conditions during her own foetal development which took place under the same social conditions. In order to discriminate between these possibilities, it would be necessary to quantify short-term patterns of hormonal variation through blood sampling and to elucidate longer term patterns by sampling individuals across multiple seasons.

### Conclusion

We explored the potential for foetal programming in a highly philopatric pinniped using a study design that made use of two neighbouring colonies that differ in social density. We found that breeding females show elevated cortisol and testosterone levels under crowded conditions. In addition, the testosterone levels of pups show density dependence and are correlated with maternal cortisol levels. While there are reasons to believe that the patterns we report could be adaptive in the context of social density, more work will be needed to elucidate how and to what extent hormones affect offspring fitness.

## Supporting Information

S1 Table Details of the 41 microsatellite loci used for the calculation of multilocus heterozygosity (see Methods for details). “Mix” denotes the PCR mastermix used and “Ta” indicates the annealing temperature used. (DOCX)

S2 Table Cortisol and testosterone values for Antarctic fur seal mother-offspring pairs. (DOCX)

## References

[1] Barker DJ, Eriksson JG, Forsen T, Osmond C. Fetal origins of adult disease: strength of effects and biological basis. Inter J Epidemiol. 2002; 31:1235–1239. 10.1093/ije/31.6.1235 12540728

[2] Gluckman PD, Hanson MA. 2005 The fetal matrix. Cambridge, UK: Cambridge University Press.

[3] Barker DJP, Winter BD, Osmund C, Margetts B, Simmonds SJ. Weight in infancy and death from ischaemic heart disease. Lancet. 1989; 2:381–383. 10.1016/s0140-6736(89)90710-1 2570282

[4] Hales CN, Barker DJ. Type 2 (non-insulin-dependent) diabetes mellitus: the thrifty phenotype hypothesis. Diabetologia. 1992; 35:595–601. 1644236 10.1007/BF00400248

[5] Bateson P, Barker D, Clutton-Brock T, Deb D, D'Udine B, Foley RA, et al Developmental plasticity and human health. Nature. 2004; 430:419–421. 15269759 10.1038/nature02725

[6] Gluckman PD, Hanson MA. Developmental origins of disease paradigm: a mechanistic and evolutionary perspective. Pediatr Res. 2004; 56:311–317. 15240866 10.1203/01.PDR.0000135998.08025.FB

[7] Kaiser S, Sachser N. The effects of prenatal social stress on behaviour: mechanisms and function. Neurosci Biobehav Rev. 2005; 29: 283–294. 15811499 10.1016/j.neubiorev.2004.09.015

[8] Fowden AL, Forhead AJ. Hormones as epigenetic signals in developmental programming. Exp Physiol. 2009; 94:607–625. 10.1113/expphysiol.2008.046359 19251980

[9] Sachser N, Hennessy MB, Kaiser S. Adaptive modulation of behavioural profiles by social stress during early phases of life and adolescence. Neurosci Biobehav Rev. 2011; 35:1518–1533. 10.1016/j.neubiorev.2010.09.002 20854842

[10] von Engelhardt N, Groothuis T. 2011 Maternal hormones in avian eggs In: Norris D, Lopez K, editors. Hormones and Reproduction of Vertebrates. Vol. 4 Amsterdam: Academic Press p. 91–128.

[11] Matthews SG. Early programming of the hypothalamo-pituitary-adrenal axis. Trends Endocrinol Metab. 2002; 13:373–380. 12367818 10.1016/s1043-2760(02)00690-2

[12] Herman RA, Jones B, Mann DR, Wallen K. Timing of prenatal androgen exposure: anatomical and endocrine effects on juvenile male and female rhesus monkeys. 2000 Horm Behav; 38:52–66. 10924287 10.1006/hbeh.2000.1608

[13] Kaiser S, Kruijver FPM, Swaab DF, Sachser N. Early social stress in female guinea pigs induces a masculinization of adult behavior and corresponding changes in brain and neuroendocrine function. Behav Brain Res. 2003a; 144:199–210. 12946610 10.1016/s0166-4328(03)00077-9

[14] Giesing ER, Suski CD, Warner RE, Bell AM. Female sticklebacks transfer information via eggs: effects of maternal experience with predators on offspring. Proc Roy Soc B. 2011; 278:1753–1759. 10.1098/rspb.2010.1819 PMC3081764 21068041

[15] Del Giudice M. Fetal programming by maternal stress: Insights from a conflict perspective. Psychoneuroendocrin. 2012; 37:1614–1629. 10.1016/j.psyneuen.2012.05.014 22694951

[16] Gleason ED, Fuxjager MJ, Oyegbile TO, Marler CA. Testosterone release and social context: When it occurs and why. Front Neuroendocrin. 2009; 30:460–469. 10.1016/j.yfrne.2009.04.009 19422843

[17] Creel S, Dantzer B, Goymann W, Rubenstein DR. The ecology of stress: effects of the social environment. Funct Ecol. 2013; 27:66–80.

[18] Rogovin K, Randall JA, Kolosova I, Moshkin M. Social correlates of stress in adult males of the great gerbil, *Rhombomys opimus*, in years of high and low population densities. Horm Behav. 2003; 43:132–139. 12614643 10.1016/s0018-506x(02)00028-4

[19] Li C, Jiang Z, Tang S, Zeng Y. Influence of enclosure size and animal density on fecal cortisol concentration and aggression in Père David’s deer stags. Gen Comp Endocrin. 2007; 151:202–209. 10.1016/j.ygcen.2007.01.014 17324429

[20] Dettmer AM, Novak MA, Meyer JS, Suomi SJ. Population density-dependent hair cortisol concentrations in rhesus monkeys (*Macaca mulatta*). Psychoneuroendocrin. 2014; 42:59–67. 10.1016/j.psyneuen.2014.01.002 PMC3959662 24636502

[21] Pearson BL, Reeder DM, Judge PG. Crowding increases salivary cortisol but not self-directed behavior in captive baboons. Am J Primatol. 2015; 77:462–467. 10.1002/ajp.22363 25598488

[22] Wingfield JC, Hegner RE, Dufty AM, Ball GF. The "Challenge Hypothesis": Theoretical Implications for Patterns of Testosterone Secretion, Mating Systems, and Breeding Strategies. Am Natural. 1990; 136:829–846.

[23] Dantzer B, Newman AEM, Boonstra R, Palme R, Boutin S, Humphries MM, et al Density Triggers Maternal Hormones That Increase Adaptive Offspring Growth in a Wild Mammal. Science. 2013; 7:1215–1217. 10.1126/science.1235765 23599265

[24] Ortiz RM, Wade CE, Ortiz CL. Effects of prolonged fasting on plasma cortisol and TH in postweaned northern elephant seal pups. Am J Physiol. 2001; 280:790–795. 10.1152/ajpregu.2001.280.3.R790 11171659

[25] Sangiao-Alvarellos S, Guzman JM, Laiz-Carrion R, Miguez JM, Del Rio MP, Mancera JM, et al Interactive effects of high stocking density and food deprivation on carbohydrate metabolism in several tissues of gilthead sea bream (*Sparus aurata*). J Exp Zool, Part A, Comp Exp Biol. 2005; 303:761–775. 10.1002/jez.a.203 16106404

[26] Bartholomew GA. A model for the evolution of pinniped polygyny. Evolution. 1970; 24:546–559. 28562995 10.1111/j.1558-5646.1970.tb01790.x

[27] Boness DJ. 1991 Determinants of mating systems in the Otariidae (Pinnipedia) In: Renouf D, editor. Behavior of pinnipeds. London: Chapman and Hall p. 1–44.

[28] Riedman M. 1990 The pinnipeds: seals, sea lions, and walruses. Berkeley, California: University of California Press.

[29] Cassini MH. The evolution of reproductive systems in pinnipeds. Behav Ecol. 1999; 10:612–616.

[30] Trillmich F, Trillmich KGK. Mating systems of pinnipeds and marine iguanas: convergent evolution of polygyny. Biol J Linn Soc. 1984; 21:209–216.

[31] Forcada J, Staniland IJ. 2008 Antarctic fur seal *Arctocephalus gazella* In: Perrin WF, Wursig B, Thewissen JGM, editors. Encyclopedia of marine mammals. 2nd edition San Diego: Academic Press p. 1325

[32] Doidge DW, McCann TS, Croxall JP. 1986 Attendance behavior of Antarctic fur seals In: Gnetry RL, Kooyman GL, editors. Fur seals: maternal strategies at land and sea. Princeton, NJ: Princeton University Press p. 102–114.

[33] Duck CD. Annual variation in the timing of reproduction in Antarctic fur seals, *Arctocephalus gazella*, at Bird Island, South Georgia. J Zool. 1990; 222:103–116.

[34] Boyd IL, McCann TS. Pre-natal investment in reproduction by female Antarctic fur seals. Behav Ecol Sociobiol. 1989; 24:377–385.

[35] Arthur B, Hindell M, Bester M, Trathan P, Jonsen I, Staniland I, et al Return Customers: Foraging Site Fidelity and the Effect of Environmental Variability in Wide-Ranging Antarctic Fur Seals. PLoS ONE. 2015; 10(3):e0120888 10.1371/journal.pone.0120888 25807082 PMC4373865

[36] Hoffman JI, Forcada J. Extreme natal philopatry in female Antarctic fur seals (*Arctocephalus gazella*). Mamm Biol. 2012; 77:71–73.

[37] Hoffman JI, Trathan PN, Amos W. Genetic tagging reveals extreme site fidelity in territorial male Antarctic fur seals *Arctocephalus gazella*. Mol Ecol. 2006; 15:3841–3847. 17032279 10.1111/j.1365-294X.2006.03053.x

[38] McCann TS, Doidge DW. 1987 Antarctic fur seal (*Arctocephalus gazella*) In: Croxall JP, Gentry RL, editors. Status, biology and ecology of fur seals. NOAA Tech Rep NMFS 51. p. 5–8.

[39] Doidge DW, Croxall JP, Baker JR. Density-dependent pup mortality in the Antarctic fur seal *Arctocephalus gazella* at South Georgia. J Zool Soc Lond. 1984; 202:449–460.

[40] Reid K, Forcada J. Causes of offspring mortality in the Antarctic fur seal, *Arctocephalus gazella*: the interaction of density dependence and ecosystem variability. Can J Zool. 2005; 83:604–609.

[41] Hofmeyr GJG, Bester MN, Pistorius PA, Mulaudzi TW, Nico de Bruyn PJ, Ramunasi JA, et al Median pupping date, pup mortality and sex ratio of fur seals at Marion Island. S Afr J Wildl Res. 2007; 37:1–8.

[42] Bonin CA, Goebel ME, Hoffman JI, Burton RS. High male reproductive skew in a low density Antarctic fur seal (Arctocephalus gazella) breeding colony. Behav Ecol. 2014; 68:597–604.

[43] Stoffel MA, Caspers BA, Forcada J, Giannakara A, Baier MC, Eberhart-Phillips LJ, et al Chemical fingerprints encode mother-offspring similarity, colony membership, relatedness, and genetic quality in fur seals. Proc Nat Acad Sci. 2015; 112: E5005–E5012. 10.1073/pnas.1506076112 26261311 PMC4568685

[44] Koren L, Mokady O, Karaskov T, Klein J, Koren G, Geffen E. A novel method using hair for determining hormonal levels in wildlife. Anim Behav. 2002; 63:403–406.

[45] Davenport MD, Tiefenbacher S, Lutz CK, Novak MA, Meyer JS. Analysis of endogenous cortisol concentrations in the hair of rhesus macaques. Gen Comp Endocrin. 2006; 147:255–261. 10.1016/j.ygcen.2006.01.005 16483573

[46] Macbeth BJ, Cattet MRL, Obbard ME, Middel K., Janz DM. Evaluation of hair cortisol concentration as a biomarker of long-term stress in free-ranging polar bears. Wildl Soc Bull. 2012; 36:747–758.

[47] Hoffman JI, Boyd ILB, Amos W. Exploring the relationship between parental relatedness and male reproductive success in the Antarctic fur seal *Arctocephalus gazella*. Evolution. 2004; 58:2087–2099. 15521464 10.1111/j.0014-3820.2004.tb00492.x

[48] Hoffman JI, Forcada J, Trathan PN, Amos W. Female fur seals show active choice for males that are heterozygous and unrelated. Nature. 2007; 445:912–914. 17287726 10.1038/nature05558

[49] Forcada J, Hoffman JI. Climate change selects for heterozygosity in a declining fur seal population. Nature. 2014; 511:462–465. 10.1038/nature13542 25056064

[50] Tiira K, Laurila A, Peuhkuri N, Piironen J, Ranta E, Primmer CR. Aggressiveness is associated with genetic diversity in landlocked salmon (*Salmo salar*). Mol Ecol. 2003; 12:2399–2407. 12919477 10.1046/j.1365-294x.2003.01925.x

[51] Välimäki K, Hinten G, Hanski I. Inbreeding and competitive ability in the common shrew (*Sorexaraneus*). Behav Ecol Sociobiol. 2007; 61:997–1005.

[52] Gentry RL, Holt JR. 1982 Equipment and Techniques for Handling Northern Fur Seals U.S. Department of Commerce National Oceanic and Atmospheric Administration, National Marine Fisheries Service, Seattle, USA p. 1–15.

[53] Hoffman JI, Boyd ILB, Amos W. Male reproductive strategy and the importance of maternal status in the Antarctic fur seal *Arctocephalus gazella*. Evolution. 2003; 57:1917–1930. 14503632 10.1111/j.0014-3820.2003.tb00598.x

[54] Aparicio JM, Ortego J, Cordero PJ. What should we weigh to estimate heterozygosity, alleles or loci? Mol Ecol. 2006; 15:4659–4665. 17107491 10.1111/j.1365-294X.2006.03111.x

[55] Coltman DW, Pilkington JG, Smith JA, Pemberton JM. Parasite-mediated selection against inbred Soay sheep in a free-living, island population. Evolution. 1999; 53:1259–1267. 28565537 10.1111/j.1558-5646.1999.tb04538.x

[56] Venables WN, Ripley BD. Modern applied statistics with S. Springer, 2002; 4th edition

[57] Crawley MJ. 2008 The R book. Chichester, UK: Wiley.

[58] Zuur A, Ieno EN, Walker N, Saveliev AA, Smith GM. 2009 Mixed effects models and extensions in Ecology with R. New York: Springer

[59] R Development Core Team. 2014 R: A language and environment for statistical computing. Vienna, Austria: R Foundation for Statistical Computing http://www.r-project.org/.

[60] Kapoor A, Dunn E, Kostaki A, Andrews MH, Matthews SG. Fetal programming of hypothalamo-pituitary-adrenal function: prenatal stress and glucocorticoids. J Physiol. 2006; 572:31–44. 16469780 10.1113/jphysiol.2006.105254 PMC1779638

[61] Weinstock M. The long-term behavioural consequences of prenatal stress. Neurosci Biobehav Rev. 2008; 32:1073–1086. 10.1016/j.neubiorev.2008.03.002 18423592

[62] Dloniak SM, French JA, Holekamp KE. Rank-related maternal effects of androgens on behaviour in wild spotted hyenas. Nature. 2006; 440:1190–1193. 16641996 10.1038/nature04540

[63] Boyd IL. Individual variation in the duration of pregnancy and birth date in Antarctic fur seals: the role of environment, age, and sex of fetus. J Mammal. 1996; 77:124–133.

[64] McCann TS. Territoriality and breeding behaviour of adult male Antarctic Fur seal, *Arctocephalus gazella*. J Zool. 1980; 192:295–310.

[65] Bryan HM, Darimont CT, Paquet PC, Wynne-Edwards KE, Smits JEG. Stress and Reproductive Hormones in Grizzly Bears Reflect Nutritional Benefits and Social consequences of a Salmon Foraging Niche. PLoS ONE. 2013; 8 (11):e80537 10.1371/journal.pone.0080537 24312230 PMC3842319

[66] Sapolsky RM, Romero LM, Munck AU. How do glucocorticoids influence stress responses? Integrating permissive, suppressive, stimulatory, and preparative actions. Endocrinol Rev. 2000; 21:55–89 10.1210/edrv.21.1.0389 10696570

[67] Turnbull AV, Rivier CL. Regulation of the hypothalamic-pituitary-adrenal axis by cytokines: actions and mechanisms of action. Physiol Rev. 1999; 79:1–71. 9922367 10.1152/physrev.1999.79.1.1

[68] Boonekamp JJ, Ros AHF, Verhulst S. Immune activation suppresses plasma testosterone level: a meta-analysis. Biol Lett. 2008; 4:741–744. 10.1098/rsbl.2008.0347 18700197 PMC2614147

[69] Staniland IJ, Boyd IL. Variation in the foraging location of Antarctic fur seals (Arctocephalus gazella) and the effects on diving behaviour. Mar Mamm Sci. 2003: 19:331–343.

[70] Spitz J, Becquet V, Rosen DAS, Trites AW. A nutrigenomic approach to detect nutrinional stress from one expression in blood samples drawn from Steller sea lions. Comp Biochem Physiol. 2015; 187:214–223. 10.1016/j.cbpa.2015.02.006 25700740

[71] Tiira K, Laurila A, Enberg K, Piironen J, Aikio S, Ranta E. Do dominants have higher heterozygosity? Social status and genetic variation in brown trout, *Salmo trutta*. Behav Ecol Sociobiol. 2006; 59:657–665.

[72] Zgaga L, Vitart V, Hayward C, Kastelan D, Polašek O, Jakovljevic M, et al Individual multi-locus heterozygosity is associated with lower morning plasma cortisol concentrations. Europ J Endocrinol. 2013; 169:59–64. 10.1530/EJE-12-0916 23636447

[73] Comin A, Tidu L, Cornacchia G, Cappa A, Renaville B, Prandi A. Neonatal period and hair cortisol in cattle as a marker of stress In: Harapin I, Kos J, editors. Proceedings of the XVI Congress of the Mediterranean Federation for Health and Production of Ruminants. 26 4 2008 Zadar, Croatia p. 221–225.

[74] Kapoor A, Lubach G, Hedman C, Ziegler TE, Coe CL. Hormones in infant rhesus monkeys’ (Macaca mulatta) hair at birth provide a window into the fetal environment. Pedr Res. 2014; 75:476–481 10.1038/pr.2014.1 PMC3961505 24418932

[75] Liggins GC. The role of the hypothalamic-pituitary-adrenal axis in preparing the fetus for birth. Am J Obstetr Gynecol. 2000; 182:475–477. 10.1016/s0002-9378(00)70241-9 10694355

[76] Andrews MH, Kostaki A, Setiawan E, McCabe L, Owen D, Banjanin S, Matthews SG. Developmental regulation of the 5-HT7 serotonin receptor and transcription factor NGFI-A in the fetal guinea-pig limbic system: influence of GCs. J Physiol. 2004; 555:659–670. 14724213 10.1113/jphysiol.2003.056705 PMC1664865

[77] Fairbanks L. A., Jorgensen M. J., Bailey J. N., Bridenthal S. E., Grzywa R., and Laudenslager M. L.. Heritability and genetic correlation of hair cortisol in vervet monkeys in low and higher stress environments. Psychoneuroendocrin. 2011; 36:1201–1208. 10.1016/j.psyneuen.2011.02.013 PMC3125414 21411232

[78] Karlén J, Frostell A, Theodorsson E, Faresjö T, Ludvigsson J. Maternal influence on child HPA axis: a prospective study of cortisol levels in hair. Pediatr. 2013; 132:1333–1340. 10.1542/peds.2013-1178 24101769

[79] Burton PJ, Waddell BJ. Dual function of 11-hydroxysteroid dehydrogenase in placenta: modulating placental glucocorticoid passage and local steroid action. Biol Reprod. 1999; 60:234–240. 9915986 10.1095/biolreprod60.2.234

[80] Welberg LA, Thrivikraman KV, Plotsky PM. Chronic maternal stress inhibits the capacity to up-regulate placental 11beta-hydroxysteroid dehydrogenase type 2 activity. J Endocrinol. 2005; 186:7–12. 10.1677/joe.1.06374 16135661

[81] Kaiser S, Heemann K, Straub RH, Sachser N. The social environment affects behaviour and androgens, but not cortisol in pregnant female guinea pigs. Psychoneuroendocrin. 2003b; 28:67–83. 10.1016/s0306-4530(02)00010-0 12445837

[82] Mazur A, Susman EJ, Edelbrock S. Sex difference in testosterone response to a video game contest. Evol Hum Behav. 1997; 18:317–326.

[83] Powell LH, Lovallo WR, Matthews KA, Meyer P, Midgley AR, Baum A, et al Physiologic Markers of Chronic Stress in Premenopausal, Middle-Aged Women. Psychosom Med. 2002; 64:502–509. 12021424 10.1097/00006842-200205000-00015

[84] Groothuis T, Müller W, von Engelhardt N, Carere C, Eising C. Maternal hormones as a tool to adjust offspring phenotype in avian species. Neurosci Biobehav Rev. 2005; 29:329–352. 15811503 10.1016/j.neubiorev.2004.12.002

[85] Meylan S, Miles DB, Clobert J. Hormonally mediated maternal effects, individual strategy and global change. Phil Trans Roy Soc B. 2012; 367:1647–1664. 22566673 10.1098/rstb.2012.0020 PMC3350661

[86] Sheriff MJ, Love OP. Determining the adaptive potential of maternal stress. Ecol Lett. 2013; 16:271–280 10.1111/ele.12042 23205937

[87] Sapolsky RM. The endocrine stress-response and social status in the wild baboon. Horm Behav. 1982; 16:279–292. 6890939 10.1016/0018-506x(82)90027-7

[88] Wingfield JC, Deviche P, Sharbaugh S, Astheimer LB, Holberton R, Suydam R, Hunt K. Seasonal changes of the adrenocortical responses to stress in redpolls, *Acanthis flammea*, in Alaska. J Exp Zool. 1994; 270:372–380.

[89] Lidgard DC, Boness DJ, Bowen WD, McMillan JI. The implications of stress on male mating behavior and success in a sexually dimorphic polygynous mammal, the grey seal. Horm Behav. 2008; 53:241–248. 18021775 10.1016/j.yhbeh.2007.10.003

[90] Kirschbaum C, Tietze A, Skoluda N, Dettenborn L. Hair as a retrospective calendar of cortisol production-increased cortisol incorporation into hair in the third trimester of pregnancy. Psychoneuroendocrin. 2009; 34:32–37. 10.1016/j.psyneuen.2008.08.024 18947933

[91] Sheriff MJ, Dantzer B, Delehanty B, Palme R, Boonstra R. Measuring stress in wildlife: techniques for quantifying glucocorticoids. Oecologia. 2011; 166:869–887. 10.1007/s00442-011-1943-y 21344254

[92] Terwissen CV, Mastromonaco GF, Murray DL. Influence of adrenocorticotrophin hormone challenge and external factors (age, sex, and body region) on hair cortisol concentration in Canada lynx (*Lynx canadensis*). Gen Comp Endocrin. 2013; 194:162–167. 10.1016/j.ygcen.2013.09.010 24080086

[93] Scheffer VB, and Johnson AM. 1963 Molt in the northern fur seal. U. S. Fish and Wildlife Services Special Scientific Report—Fisheries No. 450; Washington, D. C

[94] Ling JK. 1970 Pelage and molting in wild mammals with special reference to aquatic forms. The Quarterly Review of Biology 45 (1): 16–54. 5446769 10.1086/406361

[95] Atkinson S. Reproductive biology of seals. Rev Reprod. 1997; 2:175–194. 9414481 10.1530/ror.0.0020175

[96] Browne P, Conley AJ, Spraker T, Ream RR, Lasley BL. Sex steroid concentrations and localization of steroidogenic enzyme expression in free-ranging female northern fur seals (*Callorhinus ursinus*). Gen Comp Endocrin. 2006; 147:175–183. 10.1016/j.ygcen.2005.12.019 16473352

[97] Katz H, Pessina P, and Franco-Trecu V. Serum progesterone concentration in female South American fur seals (*Arctophoca australis*) during the breeding season. Aqua Mamm. 2013; 39:290–295.

