# Supplementary material for: Offspring Hormones Reflect the Maternal Prenatal Social Environment: Potential for Foetal Programming?
Source: PLoS One. 2016 Jan 13;11(1):e0145352. doi: 10.1371/journal.pone.0145352 (PMC4711963; doi:10.1371/journal.pone.0145352)
Supplement: S1 Table — (DOCX) [file pone.0145352.s001.docx]

**Table S1.** Details of the 41 microsatellite loci used for the calculation of multilocus heterozygosity (see Methods for details). “Mix” denotes the PCR mastermix used and “Ta” indicates the annealing temperature used.

| Locus | References | Mix | *T*_a_ | Number of alleles |
| --- | --- | --- | --- | --- |
| Pv9 | Allen *et al*. [^1^](#_ENREF_1) | 1 | 53 | 10 |
| Hg6.1 | Allen *et al*. ^1^ | 7 | 60 | 13 |
| Hg6.3 | Allen *et al*. ^1^ | 1 | 53 | 12 |
| Hg8.10 | Allen *et al*. ^1^ | 1 | 53 | 2 |
| PvcA | Coltman *et al*. ^2^ | 1 | 53 | 7 |
| PvcE | Coltman *et al*. ^2^ | 2 | 60 | 13 |
| Aa4 | Gemmell *et al*. [^3^](#_ENREF_1) | 4 | 60 | 6 |
| Hg1.3 | Gemmell *et al*. [^3^](#_ENREF_1) | 1 | 53 | 11 |
| OrrFCB2 | Buchanan *et al*. [^4^](#_ENREF_1) | 2 | 60 | 11 |
| OrrFCB7 | Buchanan *et al*. [^4^](#_ENREF_1) | 2 | 60 | 10 |
| M11a | Hoelzel *et al*. [^5^](#_ENREF_1) | 4 | 60 | 17 |
| Lc28 | Davis *et al*. [^6^](#_ENREF_1) | 4 | 60 | 9 |
| Lw10 | Davis *et al*. [^6^](#_ENREF_1) | 2 | 60 | 15 |
| Zcc7t | Hernandez-Velazquez *et al*. ^7^ | 7 | 60 | 13 |
| ZcwCgDh1.8 | Hernandez-Velazquez *et al*. ^7^ | 3 | 60 | 9 |
| ZcwDh3.6 | Hernandez-Velazquez *et al*. ^7^ | 4 | 60 | 4 |
| ZcwCgDh4.7 | Hernandez-Velazquez *et al*. ^7^ | 3 | 60 | 13 |
| ZcCgDh5.8 | Hernandez-Velazquez *et al*. ^7^ | 6 | 60 | 11 |
| ZcwCgDh7tg | Hernandez-Velazquez *et al*. ^7^ | 3 | 60 | 12 |
| ZcwCgDhB.14 | Hernandez-Velazquez *et al*. ^7^ | 2 | 60 | 6 |
| Zcwb09 | Wolf *et al*. ^8^ | 6 | 60 | 12 |
| Zcwc03 | Wolf *et al*. [^6^](#_ENREF_1)^8^ | 6 | 60 | 11 |
| Zcwc11 | Wolf *et al*. [^6^](#_ENREF_1)^8^ | 6 | 60 | 14 |
| Zcwd02 | Wolf *et al*. [^6^](#_ENREF_1)^8^ | 3 | 60 | 13 |
| Zcwe03 | Wolf *et al*. [^6^](#_ENREF_1)^8^ | 7 | 60 | 9 |
| Ssl301 | Huebinger *et al*. [^6^](#_ENREF_1)^9^ | 3 | 60 | 14 |
| Zcwa05 | Hoffman *et al*. [^7^](#_ENREF_1)^0^ | 5 | 60 | 14 |
| Zcwb07 | Hoffman *et al*. [^7^](#_ENREF_1)^0^ | 1 | 53 | 11 |
| Zcwc01 | Hoffman *et al*. [^7^](#_ENREF_1)^0^ | 2 | 60 | 11 |
| Zcwe04 | Hoffman *et al*. [^7^](#_ENREF_1)^0^ | 8 | 60 | 12 |
| Zcwe12 | Hoffman *et al*. [^7^](#_ENREF_1)^0^ | 8 | 60 | 8 |
| Zcwf07 | Hoffman *et al*. [^7^](#_ENREF_1)^0^ | 4 | 60 | 9 |
| Ag1 | Hoffman *et al*. [^7^](#_ENREF_1)^1^ | 3 | 60 | 10 |
| Ag2 | Hoffman *et al*. [^7^](#_ENREF_1)^1^ | 2 | 60 | 7 |
| Ag3 | Hoffman *et al*. [^7^](#_ENREF_1)^1^ | 2 | 60 | 2 |
| Agaz2 | Hoffman [^7^](#_ENREF_1)^2^ | 1 | 53 | 8 |
| Agaz3 | Hoffman [^7^](#_ENREF_1)^2^ | 2 | 60 | 5 |
| Agaz5 | Hoffman [^7^](#_ENREF_1)^2^ | 2 | 60 | 3 |
| Agaz6 | Hoffman [^7^](#_ENREF_1)^2^ | 2 | 60 | 4 |
| Agaz10 | Hoffman [^7^](#_ENREF_1)^2^ | 2 | 60 | 11 |
| Zcwe05 | unpublished | 3 | 60 | 9 |

^1. Allen PJ, Amos W, Pomeroy PP, Twiss SD. Microsatellite variation in grey seals (Halichoerus grypus) shows evidence of genetic differentiation between two British breeding colonies. Mol Ecol. 1995; 4: 653–662.^

^2. Coltman DW, Bowen WD, Wright JM. PCR primers for harbour seal (Phoca vitulina concolour) microsatellites amplify polymorphic loci in other pinniped species. Mol Ecol. 1996; 5: 161-163.^

^3. Gemmell NJ, Allen PJ, Goodman SJ, Reed JZ. Interspecific microsatellite markers for the study of pinniped populations. Mol Ecol. 1997; 6: 661-666.^

^4. Buchanan FC, Maiers LD, Thue TD, DeMarch BGE, Stewart REA. Microsatellites from the Atlantic walrus Odobenus rosmarus rosmarus. Mol Ecol. 1998; 7: 1083-1085.^

^5. Hoelzel AR, LeBoeuf BJ, Reiter J, Campagna C. Alpha-male paternity in elephant seals. Behav Ecol Sociobiol. 1996; 46: 298-306.^

^6. Davis CS, Gelatt TS, Siniff D, Strobeck C . Dinucleotide microsatellite markers from the Antarctic seals and their use in other pinnipeds. Mol Ecol Notes. 2002; 2: 203-208.^

^7. Hernandez-Velazquez FD, Galindo-Sanchez CE, Taylor MI, De La Rosa-Velez J, Cote IM, et al. New polymorphic microsatellite markers for California sea lions (^*^Zalophus californianus^*^). Mol Ecol Notes. 2005; 5: 140-142.^

^8. Wolf JBW, Tautz D, Caccone A, Steinfartz S. Development of new microsatellite loci and evaluation of loci from other pinniped species for the Galapagos sea lion (^*^Zalophus californianus wollebaeki^*^). Con Gen. 2005;^

^9. Huebinger RM, Louis Jr EE, Gelatt T, Rea LD, Bickham JW (2007) Characterization of eight microsatellite loci in Steller sea lions (Eumetopias jubatus). Mol Ecol Notes. 2007; 7: 1097-1099.^

^10. Hoffman JI, Steinfartz S, Wolf JBW. Ten novel dinucleotide microsatellite loci cloned from the Galápagos sea lion (^*^Zalophus californianus wollebaeki^*^) are polymorphic in other pinniped species. Mol Ecol Notes. 2007; 7: 103–105.^

^11. Hoffman JI, Dasmahapatra KK, Nichols HJ. Ten novel polymorphic dinucleotide microsatellite loci cloned from the Antarctic fur seal Arctocephalus gazella. Mol Ecol Res. 2008; 8: 459–461.^

^12. Hoffman JI. A panel of new microsatellite loci for genetic studies of Antarctic fur seals and other otariids. Conserv Gen. 2009; 10: 989–992.^
