# Supplementary material for: Offspring Hormones Reflect the Maternal Prenatal Social Environment: Potential for Foetal Programming?
Source: PLoS One. 2016 Jan 13;11(1):e0145352. doi: 10.1371/journal.pone.0145352 (PMC4711963; doi:10.1371/journal.pone.0145352)
Supplement: S2 Table — (DOCX) [file pone.0145352.s002.docx]

**Table S2.** Cortisol and testosterone values for Antarctic fur seal mother-offspring pairs.

| Pair ID | Description | Colony | Cortisol [pg/mg] | Testosterone [pg/mg] |
| --- | --- | --- | --- | --- |
| 5 | Mother | high | 14.64 | NA |
| 5 | Pup | high | 114.29 | NA |
| 6 | Mother | low | 8.33 | NA |
| 6 | Pup | low | 141.2 | NA |
| 7 | Mother | low | 20.25 | 12.89 |
| 7 | Pup | low | 102.09 | 13.58 |
| 8 | Mother | low | 9.35 | 8.52 |
| 8 | Pup | low | 56.99 | 8.14 |
| 9 | Mother | high | 44.39 | 20.15 |
| 9 | Pup | high | 115.94 | 15.88 |
| 10 | Mother | low | 25.29 | 8.58 |
| 10 | Pup | low | NA | 11.96 |
| 11 | Mother | low | 16.37 | 10.51 |
| 11 | Pup | low | 102.07 | 13.94 |
| 12 | Mother | low | 12.51 | 10.61 |
| 12 | Pup | low | 114.79 | 12.98 |
| 13 | Mother | low | 14.25 | 8.83 |
| 13 | Pup | low | 79.95 | 8.01 |
| 14 | Mother | low | 18.97 | 8.96 |
| 14 | Pup | low | 129.98 | 13.83 |
| 15 | Mother | high | 17.07 | 12.40 |
| 15 | Pup | high | 109.88 | 19.86 |
| 16 | Mother | low | 23.26 | 15.52 |
| 16 | Pup | low | 115.04 | 9.55 |
| 17 | Mother | low | 13.39 | 8.51 |
| 17 | Pup | low | 102.5 | 9.74 |
| 18 | Mother | low | 108.46 | 12.64 |
| 18 | Pup | low | 81.71 | 20.76 |
| 19 | Mother | high | 70.14 | 56.38 |
| 19 | Pup | high | 118.43 | 22.37 |
| 20 | Mother | low | 17.05 | 9.34 |
| 20 | Pup | low | 145.27 | 13.93 |
| 21 | Mother | low | 20.05 | 9.96 |
| 21 | Pup | low | 128.98 | 11.64 |
| 22 | Mother | low | 16.34 | 7.19 |
| 22 | Pup | low | 112.88 | 11.51 |
| 23 | Mother | low | 50.24 | 9.90 |
| 23 | Pup | low | 164.39 | 11.94 |
| 24 | Mother | low | 16.69 | 10.55 |
| 24 | Pup | low | 125.15 | 16.00 |
| 25 | Mother | low | 34.59 | 12.42 |
| 25 | Pup | low | 128.94 | 11.89 |
| 27 | Mother | high | 48.18 | 15.65 |
| 27 | Pup | high | 130.78 | 12.62 |
| 28 | Mother | high | 35.93 | 20.35 |
| 28 | Pup | high | 117.87 | 18.83 |
| 29 | Mother | high | 47.24 | 17.06 |
| 29 | Pup | high | 136.68 | 18.98 |
| 30 | Mother | low | 22.83 | 11.47 |
| 30 | Pup | low | 127.52 | 15.85 |
| 31 | Mother | low | 21.88 | 10.37 |
| 31 | Pup | low | 89.07 | 11.72 |
| 32 | Mother | low | 57.09 | 13.48 |
| 32 | Pup | low | 95.03 | 9.31 |
| 33 | Mother | low | 29.82 | 13.88 |
| 33 | Pup | low | 101.98 | 14.06 |
| 35 | Mother | low | 12.26 | 8.18 |
| 35 | Pup | low | 128.81 | 8.27 |
| 36 | Mother | low | 11.84 | 5.28 |
| 36 | Pup | low | 100.77 | 9.23 |
| 37 | Mother | high | 41.13 | 15.92 |
| 37 | Pup | high | 95.32 | 14.83 |
| 38 | Mother | low | 44.89 | 15.70 |
| 38 | Pup | low | 77.4 | 10.25 |
| 39 | Mother | low | 14.31 | 10.42 |
| 39 | Pup | low | 115.98 | 11.09 |
| 40 | Mother | high | 82.08 | 23.28 |
| 40 | Pup | high | 126.89 | 28.34 |
| 41 | Mother | high | 20.58 | 14.82 |
| 41 | Pup | high | 86.58 | 13.78 |
| 42 | Mother | high | 73.66 | 42.41 |
| 42 | Pup | high | NA | NA |
| 43 | Mother | high | 79.93 | 29.46 |
| 43 | Pup | high | 73.26 | 10.20 |
| 44 | Mother | high | 58.01 | 22.88 |
| 44 | Pup | high | 125.19 | 23.24 |
| 45 | Mother | high | NA | NA |
| 45 | Pup | high | 71.6 | 13.12 |
| 46 | Mother | high | 52.17 | 21.84 |
| 46 | Pup | high | 148.93 | 22.17 |
| 47 | Mother | high | 56.46 | 36.46 |
| 47 | Pup | high | 109.71 | 17.43 |
| 48 | Mother | high | 54.17 | 23.34 |
| 48 | Pup | high | 94.3 | 17.00 |
